# Mapping Neuronal Circuits Regulating Temperature Preference and Sleep in Drosophila Alongside High‐throughput Epac Imaging of Synaptosome Subtypes

**DOI:** 10.1002/alz70855_097151

**Published:** 2025-12-23

**Authors:** Vikram Simhambhatla, Paul Shaw

**Affiliations:** ^1^ Washington University School of Medicine, St. Louis, MO, USA

## Abstract

**Background:**

Sleep and temperature regulation are fundamental biological processes critical for health and survival across species. Despite their significance, the interplay between these systems remains poorly understood. Recent findings suggest overlapping neural circuits regulate both, indicating an interdependence with broad neurobiological implications. Neurodegenerative diseases, including Alzheimer's disease (AD), which are associated with impaired thermoregulation and sleep disruption, underscore the importance of understanding these mechanisms. Identifying these connections is vital for uncovering molecular pathways underlying AD symptoms and developing interventions for temperature and sleep dysregulation.

**Method:**

Our research used *Drosophila melanogaster* (fruit flies) as a model to better elucidate the relationship between sleep and thermoregulation. Two complementary strategies were implemented. First, a minimally invasive sleep‐fragmentation protocol briefly woke flies every 15 minutes, disrupting deep sleep without impairing memory or activating compensatory sleep homeostasis mechanisms. Second, genetic tools were used, including RNAi knockdown of the pigment dispersing factor receptor (Pdfr) in specific neural circuits and targeted expression of Alzheimer's‐related genes, such as Aβ‐Arctic, to explore neuronal contributions to sleep and temperature regulation. Behavioral assays assessed temperature preference under various sleep disruption protocols, including sleep deprivation, social jet lag, and sleep fragmentation, to better understand AD‐related pathology.

**Result:**

*Drosophila* subjected to sleep deprivation or fragmentation exhibited a significant increase in thermal preferences towards warmer temperatures, suggesting disrupted sleep alters thermoregulatory behaviors. Sleep fragmentation effectively disrupted deep sleep stages without impairing memory, reinforcing its utility for studying sleep regulation and providing a model for AD‐related dysfunction. RNAi‐mediated knockdown of Pdfr in clock neurons prevented the temperature preference shifts caused by sleep fragmentation, implicating these neural circuits in the behavior. Social jet lag induced persistent changes in temperature preference, highlighting the sensitivity of thermoregulation to AD‐related disruptions in circadian systems.

**Conclusion:**

Our findings highlight that the neural circuits governing temperature preference and sleep overlap significantly, revealing their functional interdependence. Introducing Alzheimer's genes broadens our understanding of the underlying molecular mechanisms of impaired thermoregulation and sleep in neurodegenerative diseases. By leveraging behavioral assays and *Drosophila* genetics, this research provides key insights into conserved mechanisms and potential therapies for addressing sleep disorders and thermoregulatory dysfunctions in AD.

